# Post Flywheel Squat Potentiation of Vertical and Horizontal Ground Reaction Force Parameters during Jumps and Changes of Direction

**DOI:** 10.3390/sports9010005

**Published:** 2021-01-05

**Authors:** Stuart A. McErlain-Naylor, Marco Beato

**Affiliations:** School of Health and Sports Sciences, University of Suffolk, Ipswich IP4 1QJ, UK; M.Beato@uos.ac.uk

**Keywords:** PAPE, countermovement jump, standing broad jump, change of direction, PAP, isoinertial, eccentric overload, kinetic, power, rate of force development

## Abstract

(1) Background: The aim of the study was to determine the post-activation performance enhancement (PAPE) of vertical and horizontal ground reaction force parameters during jumps and change of direction following flywheel squat exercise using two different flywheel inertias. (2) Methods: Eleven male athletes performed a countermovement jump (CMJ), standing broad jump (SBJ), and “modified 505” change of direction (COD) in a control condition and 6 minutes following three sets of six repetitions of flywheel half squats at one of two inertias (0.029 kg·m^2^ and 0.061 kg·m^2^). Peak directional ground reaction force, power, and rate of force development were calculated for each test. (3) Results: Higher inertia flywheel squats were able to acutely enhance CMJ peak vertical force (Bayes Factor (BF_10_) = 33.5, *very strong*; δ = 1.66; CI: 0.67, 2.70), whereas lower inertia flywheel squats were able to acutely enhance CMJ peak vertical power (BF_10_ = 3.65, *moderate*; δ = 0.93; CI: 0.11, 1.88). The vertical squat exercise induced no PAPE effect on resultant SBJ or horizontal COD ground reaction force parameters, nor were any differences observed between the inertias. (4) Conclusions: Researchers and practitioners should consider the kinetic and kinematic correspondence of a pre-load stimulus to the subsequent sport-specific activity (i.e., flywheel squat to CMJ).

## 1. Introduction

Post-activation potentiation is an acute improvement in muscular contractile performance following a pre-load stimulus such as a resistance exercise protocol of maximal or submaximal loads [1,2]. Several explanatory mechanisms have been proposed, including neuromuscular and mechanical, as well as biochemical and physiological acute responses [3,4]. Currently, the most accredited theory reports that post-activation potentiation may relate to the phosphorylation of myosin regulatory light chains during a muscle contraction, leading to a greater rate of cross-bridge attachment [2]. The role of post-activation potentiation on sports performance has been recently debated due to uncertainties around the extent to which the time frames of myosin light chain phosphorylation and voluntary force enhancement overlap [5]. An alternative term, post-activation performance enhancement (PAPE), has been proposed to incorporate changes in factors such as the temperature, water content, and activation of muscle [5,6].

Previous research investigating PAPE reported acute improvements in lower limb performance following resistance exercise [4,7,8], while other studies have failed to confirm this [2,9]. These inconsistent results could relate to differences in pre-load protocol. Exercise characteristics such as volume, intensity, muscle action, and recovery between pre-load exercise and the following test are key variables known to determine the magnitude of PAPE response [3,10]. The majority of studies evaluating the acute effects of pre-load exercise have used traditional weightlifting protocols [7,11], while evidence about PAPE following flywheel resistance exercise remains limited and superficial [4,12].

The utility of flywheel devices for inducing PAPE is only recently beginning to be understood [10]. Such devices are commonly utilized to facilitate eccentric overload protocols in which the generated eccentric muscular force may exceed the maximal concentric force [13,14]. The user rotationally accelerates the flywheel (resistance due to the flywheel moment of inertia) during the concentric phase of the movement, resulting in flywheel kinetic energy and inertial torque that imparts high linear resistance during the subsequent eccentric phase of the movement [15,16]. Despite the observed acute improvements in whole-body dynamic performance, a thorough analysis of the PAPE response in specific directional kinetic parameters following flywheel exercise has not been conducted. The impulse-momentum relationship necessitates that an improvement in jump distances or change of direction (COD) time must be associated with an increase in ground reaction force impulse in the appropriate direction. What remains less clear is the PAPE effect on peak force, peak power, and/or peak rate of force development during specific vertically and/or horizontally dominant activities [4].

In the only investigations of such parameters to date, flywheel squat exercise induced increases in countermovement jump (CMJ) peak force and peak power [4]. Similarly, flywheel squats acutely enhanced peak isokinetic eccentric knee flexion torque [17]. Evidence of an effect on peak concentric knee flexion and extension torques has been inconsistent [4,17]. No previous studies have reported the effect of flywheel PAPE protocols on CMJ peak rate of force development. Moreover, the flywheel-induced PAPE effects on such ground reaction force measures as peak force, peak power, and peak rate of force development are yet to be investigated in horizontally dominant activities such as the COD or activities requiring concurrent horizontal and vertical force application such as the standing broad jump (SBJ). A recent investigation comparing protocols with different flywheel squat inertias reported PAPE effects on CMJ, SBJ, and COD performance outcomes, but no difference in the magnitude of responses between inertias [18]. It is currently unknown whether the PAPE effects with each inertia were achieved through the same or different kinetic mechanisms. For example, there may be a specificity of transfer from the force–velocity region occupied during the pre-load exercise (due to exercise inertia) to the subsequent kinetic PAPE response (e.g., peak force, peak power, and/or peak rate of force development) [19,20,21,22].

The aim of the present study was to determine the PAPE effects of flywheel half squat exercise with two different flywheel inertias on vertical and horizontal ground reaction force parameters during jumps and changes of direction. It was hypothesized that flywheel half squat exercise would induce a PAPE effect on peak force and peak rate of force development during CMJ, SBJ, and COD, as well as on peak power during CMJ and SBJ. No *a priori* hypothesis was made regarding the effect of flywheel inertia on these effects due to the lack of prior evidence.

## 2. Materials and Methods

### 2.1. Participants

An a priori power analysis (G*Power version 3.1.9.7, Heinrich Heine University Düsseldorf, Germany) revealed that 10 participants would provide an 85% chance of achieving α = 0.05 in a frequentist 2 × 2 repeated measures analysis of variance, assuming an effect size of 0.40 (from a previous PAPE effect on CMJ peak power 5 min after flywheel squat exercise [4]) and a high correlation (*r* = 0.8) between repeated measures. Multiplication by a Bayesian inflation factor of 1.084 resulted in the requirement for 11 participants [23]. Eleven male recreational team sports athletes (22 ± 2 years; 82.6 ± 12.5 kg) participated in this investigation, each with more than 10 years of experience in their sport and currently participating in at least one resistance training session per week. All participants trained and competed at least twice per week and were free from injury or illness. Each was familiar with the flywheel squat exercise and the CMJ, SBJ, and “modified 505” COD tests, as recently suggested [24]. The study was approved by the ethical advisory committee of the University of Suffolk. Participants were informed of the benefits and risks of the investigation prior to signing informed consent.

### 2.2. Design

A within-participants randomized controlled crossover design was utilized. Each participant attended the laboratory on 5 separate occasions, separated by at least 48 h of recovery to avoid any possible transient fatigue due to repeated efforts. The 5 testing sessions were randomly allocated to reduce learning effects. Participants maintained their normal nutritional intake during the experimental period. Alcohol and caffeine were not permitted prior to the experimental sessions but hydration was allowed during the sessions. The 5 sessions were as follows in a randomized order: (1) control condition (no pre-load exercise) followed by CMJ, SBJ, and COD; (2) lower inertia flywheel half squats followed by jumps (CMJ and SBJ); (3) lower inertia followed by COD; (4) higher inertia flywheel half squats followed by jumps; (5) higher inertia followed by COD.

### 2.3. Procedures

A standardized warm-up consisting of 5 min of cycling at a constant power on an ergometer (Sport Excalibur lode, Groningen, The Netherlands) was performed at the beginning of each testing session [4]. Mobilization was performed immediately after the cycling for a duration of 3 min, consisting of dynamic movements mimicking the exercise (e.g., half squat) and dynamic hip, knee, and ankle movements. In the control session, a maximal CMJ, SBJ, and COD were performed in a randomized order following the warm-up. The warm-up in each of the other sessions was followed by 3 sets of 6 flywheel ergometer (D11 Full, Desmotec, Biella, Italy) half squat repetitions, with 2 minutes of passive recovery between sets [4]. This multi-set exercise protocol has previously been utilized to successfully induce PAPE in similar cohorts of participants [4,25]. The flywheel half squats were followed after 6 min of passive recovery by maximal efforts of either 1 CMJ and 1 SBJ (order randomized), or 1 COD only, depending on the session. This timing (6 min) after the pre-load exercise has previously been shown to characterize the optimal PAPE response following such exercise [18]. Randomization of sessions, as well as tests within a session, minimized potential confounding effects of training, learning, or fatigue.

### 2.4. Flywheel Exercise

Participants were instructed to perform the concentric phase of the flywheel half squats with maximal velocity, and the eccentric phase to approximately 90° of knee flexion as in previous similar successful acute interventions [12,17,18,25]. The combined load of the ergometer plus flywheel was 1 “large” disc and 1 “medium” disc (mass = 3.0 kg; moment of inertia = 0.029 kg·m^2^) during lower inertia exercise, and 1 “Pro” disc (mass = 6.0 kg; moment of inertia = 0.061 kg·m^2^) during higher inertia exercise. These moments of inertia were used to generate PAPE effects on CMJ, SBJ, and COD performance outcomes in a previous investigation [18].

### 2.5. Physical Tests

Ground reaction force parameters during the CMJ, SBJ, and COD were assessed using a force platform (Kistler, Winterthur, Switzerland; 900 × 600 mm; 1000 Hz). CMJs were performed for maximum height from an upright stationary starting position with a self-selected depth, and hands on hips to avoid influence of arm swing [26]. Bilateral SBJs for maximum anterior distance were performed from an upright stationary starting position with a self-selected depth and arm swing. COD (aiming for minimum completion time) was tested via a dominant leg unilateral 180° turn separating 2 × 5 m sprints [27], commonly referred to as a “modified 505”. COD trials started on the “Go” command from a standing position, with the front foot up to the measured starting mark [28].

### 2.6. Dependent Variables

All kinetic parameters were calculated in MATLAB (Version R2020a, The MathWorks Inc., Natick, MA, USA) from ground reaction force data in the primary direction of performance determination. For CMJ, peak vertical force, power, and rate of force development were calculated. For SBJ, peak resultant (of anterior–posterior and vertical) force, power, and rate of force development were calculated. For COD, peak anterior-posterior force and rate of force development were calculated. Thus, eight kinetic parameters were determined for each of control, following lower inertia, and following higher inertia. Peak force was determined as the maximum positive (vertical or anterior) ground reaction force at any time point during ground contact prior to take-off. Peak power was determined as the maximum dot product of force and velocity (velocity calculated via the impulse method) at any time point during ground contact prior to take-off. Peak rate of force development was determined as the maximum positive rate of ground reaction force development at any time point during ground contact prior to take-off (which was calculated as the average rate of development over a 50 ms period using a rolling mean method) [29]. Ground contact was identified using a 10 N vertical ground reaction force threshold.

### 2.7. Statistical Analyses

All statistical analyses were performed within JASP (University of Amsterdam, The Netherlands) software version 0.9.2.0. Data were presented as mean ± SD. A fully Bayesian inferential statistical approach was used to provide probabilistic statements (see [30] for an introduction). Each analysis was conducted with the JASP-default “noninformative” prior (Cauchy distribution, 0.707) due to the lack of strong a priori evidence [31]. Bayesian repeated measures ANOVA was used to evaluate the effects of time (within: control vs. 6 min) and inertia (between: lower vs. higher inertia) on the 8 kinetic parameters describing CMJ, SBJ, and COD performance. Bayes factor (BF_10_) was reported to indicate the strength of the evidence and interpreted as follows: 1/3 < anecdotal ≤ 3; 3 < moderate ≤ 10; 10 < strong ≤ 30; 30 < very strong ≤ 100; extreme > 100 [32]. Evidence for the alternative hypothesis (H_1_) was set as BF_10_ > 3, and evidence for the null hypothesis (H_0_) was set as BF_10_ < 1/3. Estimates of median standardized effect size (δ) and 95% credible interval (CI) were calculated [32]. Markov Chain Monte Carlo with Gibbs sampling was used to make inferences (10,000 samples). δ was interpreted by Cohen as follows: trivial < 0.2; 0.2 ≤ small < 0.6; 0.6 ≤ moderate < 1.2; 1.2 ≤ large < 2.0; very large ≥ 2.0 [33].

## 3. Results

Meaningful PAPE (within: 6 min vs. control) effects were reported for CMJ peak force (BF_10_ = 294; extreme), CMJ peak power (BF_10_ = 7.12; moderate), and CMJ peak rate of force development (BF_10_ = 4.35; moderate). No meaningful PAPE effects were reported for any SBJ or COD parameters (within: 0.247 ≤ BF_10_ ≤ 0.712; Table 1). No meaningful differences between exercise inertias (between: 0.223 ≤ BF_10_ ≤ 0.569) or interactions between time and inertia (0.100 ≤ BF_10_ ≤ 0.883) were reported (Table 1). Bayesian post hoc comparisons of the PAPE effect (control vs. 6 min; Table 2) reported meaningful differences in CMJ peak force following higher inertia (BF_10_ = 33.5; very strong; δ = 1.656; CI: 0.666, 2.695) and CMJ peak power following lower inertia (BF_10_ = 3.65; moderate; δ = 0.932; CI: 0.110, 1.877). No other post hoc comparisons reported meaningful differences (0.689 ≤ BF_10_ ≤ 2.738).

## 4. Discussion

This study investigated the PAPE effects on specific directional ground reaction force parameters following flywheel half squat exercise and compared such effects between different flywheel inertias. The present study reported a meaningful PAPE effect on CMJ peak vertical force following higher inertia (*very strong* likelihood) but not lower inertia flywheel squats. Contrastingly, a meaningful PAPE effect on CMJ peak power was reported following lower inertia (*moderate*) but not higher inertia flywheel squats. A meaningful (*moderate*) overall PAPE effect on CMJ peak rate of force development was reported. No meaningful differences between inertias were reported. Thus, this study reports for the first time that higher and lower inertia flywheel squats can differentially enhance CMJ peak force and peak power, respectively.

The observation of PAPE in all vertical kinetic parameters measured during the CMJ supports the present study’s hypothesis that flywheel half squat exercise can induce a PAPE effect on peak force, peak power, and peak rate of force development during a CMJ. This further supports the findings of previous studies reporting flywheel-induced PAPE effects on CMJ performance (e.g., jump height) and associated kinetic (e.g., peak vertical force and power) measures [4,12]. However, the lack of a PAPE effect on horizontal kinetic parameters during the COD or resultant kinetic parameters during the SBJ suggests that the kinetic response to a flywheel pre-load exercise relates to its specific directional loading nature (e.g., vertical loading relative to the participant during a squat exercise and a CMJ) [34]. The fact that acute performance enhancements have previously been observed in SBJ and COD following flywheel squats [12,18] suggests that further research is needed to verify the hypothesis of an association between specific pre-load directional loading nature and subsequent task performance or ground reaction force application. It may be supposed that any such effect relates more to dynamic correspondence (e.g., the corresponding regions of muscle force-length and force-velocity curves occupied during the activities) than a simple dichotomy of vertical or horizontal exercises relative to the participant.

A previous study investigating overall performance in the same tasks as the current study reported no meaningful effect of flywheel inertia on magnitude of PAPE response [18]. The present study investigated this effect in more detail, reporting on specific kinetic parameters, and again found no overall meaningful difference between flywheel inertias. Post hoc analysis of specific effects revealed contrasting meaningful PAPE effects on CMJ peak vertical force following higher but not lower inertia exercise, and on CMJ peak vertical power following lower but not higher inertia exercise. These initial results indicate the possibility of a relationship between flywheel exercise intensity and the mechanism of subsequent acute performance enhancement. The results suggest that practitioners can use three sets of six flywheel squats 6 min prior to countermovement jumps within training interventions to acutely enhance specific kinetic parameters within the jump. A recent study reported that increases in flywheel inertia are associated with decreases in peak and mean velocities during the concentric and eccentric phases of the flywheel squat but had no significant effect on peak concentric or eccentric power [22]. The study recommended the use of velocity-based flywheel resistance training [35], using peak concentric velocity to individualize exercise prescription. The present study suggests there may exist a specificity of transfer from the force–velocity region occupied during the pre-load exercise (due to flywheel inertia) to the subsequent kinetic PAPE response (e.g., peak force, peak power, and/or peak rate of force development). For example, the likely greater forces generated at higher inertias (prescribed via lower target velocities) may transfer more to acute improvements in countermovement jump peak force, whilst the greater velocities at lower inertias may transfer more to acute improvements in countermovement jump peak power [22,36]. Further research is needed to assess the efficacy of such individualized longitudinal interventions (e.g., in response to individual athlete force or velocity deficiencies) [37,38].

Whilst this study supports previous knowledge that PAPE is observed following a recovery period (e.g., 6 min) [2], it does not attempt to determine whether a shorter or longer passive recovery (e.g., 3 or 10 min) may be more effective to enhance kinetic PAPE response during sport-specific tasks. Future studies using different recovery times may be useful to clarify whether different intensities could be more effective at inducing quicker or slower PAPE response onsets. If this were the case then time windows may be altered for the specific pre-load strategy adopted [39]. Moreover, the interpretation of the current findings could imply that protocols using horizontally loaded pre-load exercises relative to the participant may exhibit the opposite direction-specificity of PAPE response (e.g., greater transfer to horizontal kinetic parameters). Further research is needed to confirm or deny this rationale, as well as to similarly investigate the effect of squat depth on observed responses [40]. The use of electromyography, beyond the scope of the current study, would enable relationships between pre-load exercise and subsequent muscle activation to be established. This may provide insight into the underlying neurophysiological mechanisms. Finally, this research enrolled male team sports athletes and thus wider generalization to alternative samples (e.g., females, professional athletes, resistance training experts) cannot be inferred. Such groups may exhibit different PAPE response characteristics, especially given relationships between muscular capabilities and PAPE magnitude [39,41,42]. Portability, access, and adherence to flywheel protocols should be considered by strength and conditioning practitioners before implementing flywheel-based training interventions.

## 5. Conclusions

The vertically dominant flywheel squat pre-load exercise induced increases in peak vertical force, power, and rate of force development during CMJ, but no such increases in resultant or horizontal kinetic parameters during the SBJ or COD. As such, practitioners should consider the directional nature and kinetic characteristics of the required acute kinetic response in order to optimize the programming of PAPE-based training or competition interventions. Meaningful PAPE effects were reported on CMJ peak vertical force following higher inertia but not lower inertia flywheel squats, and on CMJ peak vertical power following lower inertia but not higher inertia flywheel squats. Therefore, higher inertia flywheel exercise may be more effective at enhancing peak force, and lower inertias may be more effective at enhancing peak power, during subsequent CMJ. Since this is the first study to have analyzed such effects, future research is needed before drawing final conclusions.

## Figures and Tables

**Table 1:** Within (time: post-activation performance enhancement (PAPE)) and between (inertia) effects on directional kinetic parameters describing countermovement jump (CMJ), standing broad jump (SBJ), and change of direction (COD) performance 6 min following flywheel half squat exercise with lower (0.029 kg·m^2^) and higher (0.061 kg·m^2^) inertia (*n* = 11).

Variable	Inertia (kg·m^2^)	Control	6 min	BF_10_ within (Time)	Assessment	BF_10_ between (Inertia)	Assessment
CMJ PF (BW)	0.029	2.27 ± 0.19	2.40 ± 0.23	293.8	*extreme* H_1_	0.392	*anecdotal*
	0.061		2.44 ± 0.24				
CMJ PP (W·kg^−1^)	0.029	49.94 ± 4.50	52.73 ± 6.07	7.122	*moderate* H_1_	0.417	*anecdotal*
	0.061		51.40 ± 5.37				
CMJ PRFD (BW·s^−1^)	0.029	10.06 ± 2.59	11.42 ± 4.39	4.354	*moderate* H_1_	0.569	*anecdotal*
	0.061		12.91 ± 4.92				
SBJ PF (BW)	0.029	2.55 ± 0.26	2.52 ± 0.30	0.257	*moderate* H_0_	0.242	*moderate* H_0_
	0.061		2.54 ± 0.25				
SBJ PP (W·kg^−1^)	0.029	44.40 ± 4.85	43.37 ± 4.50	0.263	*moderate* H_0_	0.275	*moderate* H_0_
	0.061		44.14 ± 5.54				
SBJ PRFD (BW·s^−1^)	0.029	11.70 ± 3.82	12.47 ± 3.39	0.247	*moderate* H_0_	0.269	*moderate* H_0_
	0.061		11.64 ± 4.62				
COD PF (BW)	0.029	1.21 ± 0.24	1.18 ± 0.23	0.324	*moderate* H_0_	0.223	*moderate* H_0_
	0.061		1.17 ± 0.18				
COD PRFD (BW·s^−1^)	0.029	19.98 ± 7.02	18.58 ± 4.03	0.712	*anecdotal*	0.283	*moderate* H_0_
	0.061		17.37 ± 4.76				

PF: peak force; PP: peak power; PRFD: peak rate of force development; H_0_: evidence for the null hypothesis; H_1_: evidence for the alternative hypothesis. All kinetic parameters are vertical ground reaction force for CMJ, resultant (vertical and anterior–posterior) for SBJ, and anterior–posterior for COD.

**Table 2:** Bayesian post hoc comparisons (PAPE effect) of directional kinetic parameters describing countermovement jump (CMJ) performance between control and 6 min following lower (0.029 kg·m^2^) and higher (0.061 kg·m^2^) inertia flywheel half squat exercise (*n* = 11).

Variable	Inertia (kg·m^2^)	Control	6 min	BF_10_	Assessment	*δ*	*δ* 95% Credible Interval	Assessment
CMJ PF (BW)	0.029	2.27 ± 0.19	2.40 ± 0.23	2.738	*anecdotal*	0.854	0.037, 1.770	*moderate*
	0.061		2.44 ± 0.24	33.549	*very strong*	1.656	0.666, 2.695	*large*
CMJ PP (W·kg^−1^)	0.029	49.94 ± 4.50	52.73 ± 6.07	3.650	*moderate*	0.932	0.110, 1.877	*moderate*
	0.061		51.40 ± 5.37	0.689	*anecdotal*	0.446	−0.251, 1.279	*small*
CMJ PRFD (BW·s^−1^)	0.029	10.06 ± 2.59	11.42 ± 4.39	0.725	*anecdotal*	0.462	−0.255, 1.293	*small*
	0.061		12.91 ± 4.92	1.904	*anecdotal*	0.741	−0.046, 1.655	*moderate*

PF: peak force; PP: peak power; PRFD: peak rate of force development; δ: median standardized effect size. All CMJ kinetic parameters are vertical ground reaction force.

## Data Availability

The data presented in this study are available on reasonable request from the corresponding author.
